# Magnetic Liquid Metal (Fe‐EGaIn) Based Multifunctional Electronics for Remote Self‐Healing Materials, Degradable Electronics, and Thermal Transfer Printing

**DOI:** 10.1002/advs.201901478

**Published:** 2019-08-22

**Authors:** Rui Guo, Xuyang Sun, Bo Yuan, Hongzhang Wang, Jing Liu

**Affiliations:** ^1^ Department of Biomedical Engineering School of Medicine Tsinghua University Beijing 100084 China; ^2^ Beijing Key Laboratory of Cryo‐Biomedical Engineering and CAS Key Laboratory of Cryogenics Technical Institute of Physics and Chemistry Chinese Academy of Sciences Beijing 100190 China

**Keywords:** magnetic Fe‐EGaIn, self‐healing, thermal transfer printing, transient electronics

## Abstract

Flexible materials with the ability to be bent, strained, or twisted play a critical role in soft robots and stretchable electronics. Although tremendous efforts are focused on developing new stretchable materials with excellent stability, inevitable mechanical damage due to long term deformation is still an urgent problem to be tackled. Here, a magnetic healing method based on Fe‐doped liquid metal (Fe‐GaIn) conductive ink via a noncontact way is proposed. Further, multifunctional flexible electronics are designed with combined performances of superior remote self‐healing under magnetic field, water‐degradable, and thermal transfer printing, which attribute to three parts of the materials including Fe‐GaIn conductive ink, degradable PVA substrate, and adhesive fructose. The as‐made light emitting diodes (LED) circuit is demonstrated with both structural and functional repairing after single or multipoint damage. The self‐healing time from multipoint damage is pretty fast within 10 s. Due to the water‐soluble PVA film, the recycling process is simple via immersing into water. Through heating, the electric circuit on fructose can be transferred to other flexible substrate with high efficiency, which broadens the practical applications of the present system. The novel and multifunctional electronics hold great promise for self‐healing electronics, transient electronics, and soft robots.

## Graphical abstract

**Multifunctional flexible electronics involving integrated features of remote self‐healing, water‐degradable and thermal transfer printing** are developed. Here, a magnetic healing approach is proposed based on Fe‐doped liquid metal (Fe‐GaIn) conductive ink, which is demonstrated with both structural and functional repair under magnetic field with a magnet after single or multipoint damage.

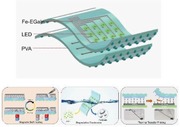

## 1 Introduction

Inspired by natural biological systems, soft and stretchable materials are extensively investigated to largely reduce the mismatch between rigid components and soft tissues, and to achieve perception capability like skin or mechanical loading such as human muscles through fulfilling various complex deformation including straining, bending, and twisting.^1^, ^2^, ^3^, ^4^, ^5^ To date, numerous efforts on creating various soft materials have mainly devoted to the development of novel materials and technologies such as construction of new conductive polymers, formation of special patterns like wavy or serpentine structure or inclusion conducive inorganic materials to high elastomers.^6^, ^7^, ^8^, ^9^ Among them, liquid metals especially arose increasing attention and are regarded as ideal materials for the next generation in applications of soft robots,^10^ wearable electronics,^11^, ^12^ and biomedical equipment.^13^, ^14^ Especially, such flexible materials exhibit huge promise as conformable electrodes,^15^, ^16^ strain sensors^17^ or reconfigurable antenna,^18^ which attributes to their superior combined features of intrinsic flowability, high electroconductivity, unique shape transformation, stimulus‐responsive properties with regard to external light, thermal, pH and chemicals, a low toxicity and vapor pressure compared to mercury etc.^19^, ^20^

Despite remarkable achievements, it is still challenging to avoid mechanical damage of flexible circuits undergo long‐term deformation and strain tension, making them vulnerable than those rigid counterparts.^21^, ^22^, ^23^, ^24^ Liquid metals exhibit unique self‐healing capability as flexible materials, e.g., liquid flow mediated reconnection and rewiring of stretchable circuits,^25^ heat stimulation and phase change triggered structure self‐healing of elastomer,^26^ and autonomous electrical self‐healing of liquid metal droplets embedded in silicone elastic materials.^27^ However, the existed issues including involvement of workforce, external energy and devices, long healing period, circuit stability as well as function recovery after healing process remain challenging. Thus, developing a method with not only structure, but also circuit function recovery capability in a simple, fast or even noncontract way through 3D field energy like magnetic tool, is highly desirable in flexible electronic areas.

Due to the high surface tension of liquid metals, liquid metals are usually considered incompatible with other materials. However, an oxide skin would form on the surface in ambient air with a thickness of about 3 nm.^28^ The metal oxide is considered as a bridge to facilitate mixture formation with other metal particles, through which the resulting materials are endowed with enhanced electrical conductivity, mechanical strength or even adhesive capability.^29^, ^30^, ^31^ Such materials with nickel particles addition are recently applied in patterning technology and applications of wearable devices with a plenty of advantages.^32^, ^33^ Thus, through iron particles mixing,^34^, ^35^ creating innovative flexible circuits with magnetic liquid metal would achieve remote response under magnetic field after damage and serve as a solution toward enhancing the long‐term performance of deformable electronics. On the other hand, the increasing electronic waste has become large sources of environmental pollution, most of which is hard to degrade.^36^, ^37^ Meanwhile, the intrinsic soft of liquid metal and the removal of the oxide skin on its surface via mechanical, chemical, or electric methods facilitate the recollection of materials.^38^ When the substrate is water‐soluble, the melting of the substrate contributes not only the dissolution, but also the recycling process via the mechanical force from the deformation of the substrate. Moreover, through transfer printing approach, liquid metal circuits could be easily reprinted on other polymers or high elastomers to broaden the applications of magnetic liquid metal materials.^39^, ^40^, ^41^

In this study, we demonstrate a magnetic liquid metal based flexible electronics with combined properties of superior remote self‐healing, water‐degradable and thermal transfer printing, which enable versatile functionalities on one flexible platform. The designed soft electronics consist of three parts including Fe‐doped liquid metal (Fe‐GaIn), degradable PVA substrate, and adhesive fructose. Each of them would fulfill one unique performance. Taking advantage of magnetic attraction of iron particles under magnet, the flowable liquid metal alloy would be driven accurately along the moving of magnet to heal the conductive lines where mechanical damage occurs in a short recovering time. Under water circumstance, the PVA substrate would dissolve and the conductive soft wires of magnetic liquid metals could be easily collected and recycled. Moreover, we found an interesting phenomenon that the transfer printing efficiency of liquid metals would be enhanced by slight heating to break the hydrogen bond of the fructose where the magnetic liquid metal contacts. This multifunctional flexible platform shows great prospects in designing future stretchable electronics, recyclable electronics, and soft robots in special needs.

## 2 Results and Discussion

### 2.1 Preparation of Magnetically Self‐Healing Liquid Meal Electronics on PVA Substrate

To fabricate magnetic liquid metal, Fe microparticles (weight of 15 g) were scattered on the surface of gallium and indium alloy (EGaIn) (weight of 100 g). Especially, in order to accelerate the dispersion of Fe microparticles into EGaIn, they were constantly agitated by a glass bar until they were completely mixed, as shown in Figure S1 (Supporting Information). The previous studies showed that the gallium‐based liquid metal was readily oxidized in the air, forming a thin oxide skin. In this process, the Fe microparticles were gradually wrapped by the gallium oxide film and were carried into the inner of EGaIn. As a result, the surface tension and liquidity of EGaIn were reduced. Meanwhile, other studies about EGaIn mixed with solid metal particles have also confirmed that the solid metal particles could reduce the liquidity of EGaIn.^29^, ^31^ Besides, the previous research has demonstrated that Fe‐EGaIn was covered by the gallium oxidation layer.^42^ In X‐ray diffraction (XRD) test, the Fe microparticles appeared in a form of Fe. No alloy of iron was detected (Figure S2, Supporting Information). Here, the packing ratio was defined as φ = mFe/mEGaln, where mFe and mEGaln represent the mass of Fe microparticles and EGaIn, respectively. Fe‐EGaIn composites with various Fe proportions were prepared with a packing ratio ranging from 0% to 25%. The electrical conductivity was tested correspondingly, which showed that the electrical conductivity was decreased with the increase of Fe proportion (Figure S3, Supporting Information). Besides, the photos of Fe‐GaIn with various Fe proportions with or without magnetic field were also captured. We can see that the liquidity of Fe‐EGaIn was decreased and the plasticity was enhanced with the addition of large amounts of Fe (Figure S4, Supporting Information). Furthermore, the material with high Fe content exhibited more sharp spine‐like structure under magnetic field and they were easy to separate from each other, which might hinder the self‐healing process. So we selected the φ = 15% Fe‐EGaIn to fabricate liquid meal electronics, and its conductivity is 1.53 × 10^6^ S m^−1^.We selected the φ = 15% Fe‐EGaIn to fabricate liquid meal electronics, and its conductivity is 1.53 × 106 S m^−1^.

**Figure 1:**
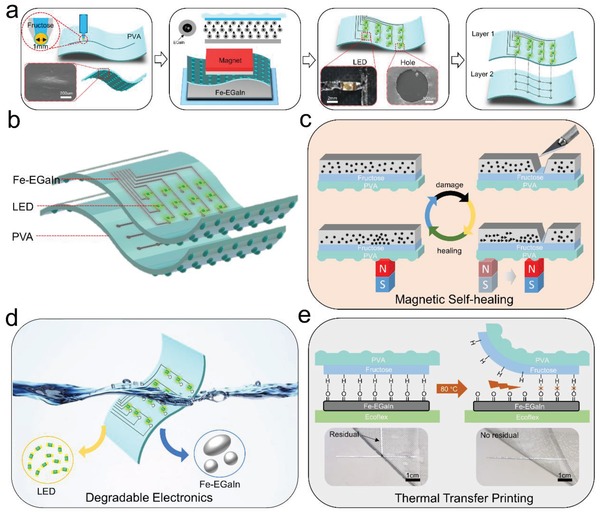
a) Preparation process of magnetic healing liquid meal electronic on PVA substrate. b) The structure of the Fe‐EGaIn based electronics. c) Schematic illustration of the healing process of Fe‐EGaIn electronics using magnetic field. d) Schematic illustration of the PVA/fructose enabled degradable and recyclable electronics. e) Schematic illustration of the hydrogen bond break enabled thermal transfer printing.

The wettability of EGaIn mixed with solid metal particles mainly depended on the morphology and interfacial chemical properties of the substrate material. For example, the semi‐liquid metal (Ni‐EGaIn) exhibited good wettability and adhesion on PMA glue but not on skin.^43^ Here, we found that the Fe‐EGaIn also showed adhesive difference on fructose and PVA film. Inspired by the phenomenon, we proposed a fabrication method of magnetic healing liquid meal electronic on PVA substrate. **Figure** **1**a illustrates the preparation process of magnetic healing liquid meal electronic on PVA substrate. At first, a PVA film with a thickness of 25 µm was applied to a glass flat sheet and the ball‐point pen filled with fructose was fixed on a mechanical arm. It was noticeable that the upper surface of PVA film was smooth, while there were equally distributed elliptic projections about 68 µm in height on its lower surface, as shown in the Figure S5 (Supporting Information). Due to these elliptic projections, there was interval between two PVA films, which helped to protect the Fe‐EGaIn lines on the lower PVA film from mechanical injury. The mechanical arm controlled the ball‐point pen to print the fructose on PVA film. Then, the upper surface of PVA film with fructose was put on the Fe‐EGaIn container, and a magnet (square, 9 cm × 9 cm) was placed on the PVA film. Under the attraction of a magnet, Fe microparticles carried the EGaIn to the upper surface of PVA film. Meanwhile, the Fe‐EGaIn was only adhered to the area of fructose, not the PVA film due to the selective adhesive behavior. Later, some electronic components (light emitting diodes (LEDs)) were integrated with the Fe‐EGaIn lines (1.5 mm width) and some holes (2 mm in diameter) were made on the top layer PVA film. Especially, in order to fix these components on top layer PVA film, a little fructose was printed on the bottom surface of these electronic components. Using the same method, a Fe‐EGaIn pattern was transfer printed on the bottom PVA film. Finally, two PVA films were glued together using fructose printed between two layers, and some Fe‐EGaIn was filled into these holes to connect the Fe‐EGaIn lines on both PVA films (Figure S6, Supporting Information). The schematic diagram in Figure 1b shows the structure of the Fe‐EGaIn based electronics.

The flexible electronics developed in this paper were mainly composed of three parts: magnetic Fe‐EGaIn, degradable PVA film, and adhesive fructose. Due to the unique properties of each material, the liquid metal electronics were capable of realizing multifunctional performances: magnetic healing, degradable electronics, and thermal transfer printing. Briefly, 1) When a moving magnetic field was applied to the Fe‐EGaIn lines, the Fe microparticles aggregated and carried the EGaIn to fill the mechanical damage gap (Figure 1c). 2) The PVA substrate and fructose were water‐soluble and the Fe‐EGaIn based electronics could be degraded in water in a short time. The electronic components (such as LEDs) and Fe‐EGaIn materials could be collected using hydrochloric acid solution (Figure 1d). 3) The hydrogen bond between liquid metal oxide layer and fructose layer could be destroyed under high temperature condition (such as 80 °C). Thus, the Fe‐EGaIn lines could be transferred with high efficiency to Ecoflex substrate at 80 °C (Figure 1e).

### 2.2 Surface Characterization of Fe‐EGaIn

**Figure 2:**
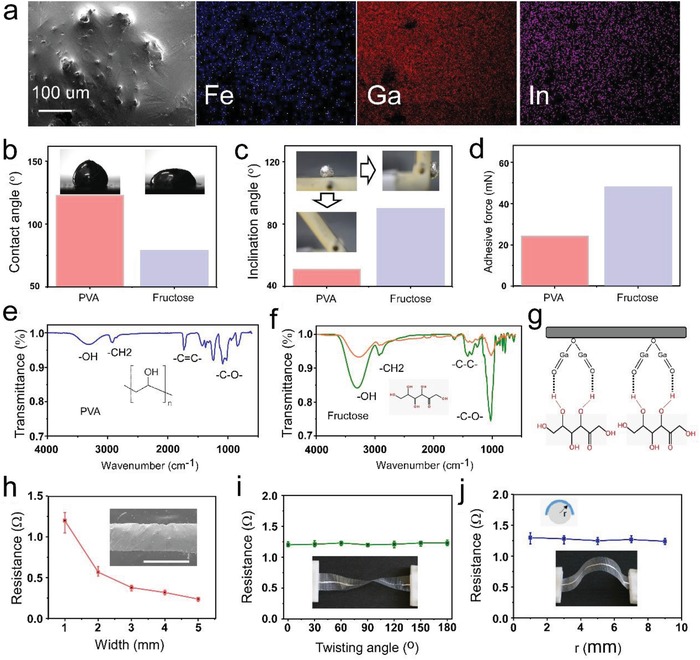
Surface characterization and electrical performance of Fe‐EGaIn electronics. a) SEM of the Fe‐EGaIn, and the related element mapping of Fe, Ga, and In, respectively. b) The contact angle of Fe‐EGaIn droplets on PVA film and fructose layer. c) The images and roll away angles of Fe‐EGaIn droplets on inclined plates with PVA and fructose, respectively. d) The adhesion force of Fe‐EGaIn with PVA and fructose. e) The molecular structure and acting functional groups of PVA. f) The molecular structure and acting functional groups of fructose (green line) and fructose covered with Fe‐EGaIn oxide layer (orange line). g) Schematic illustration of the hydrogen bonds between fructose and Fe‐EGaIn oxide layer (Ga_2_O_3_). h) The resistance of different width of Fe‐EGaIn lines (scale bar = 1 mm). i) The resistance of Fe‐EGaIn lines with twisting angles from 0° to 180°. j) The resistance of Fe‐EGaIn lines with bending radii from 1 to 9 mm.

Scanning electron micrograph (SEM) of the Fe‐EGaIn and related energy‐dispersive X‐ray spectrum (EDX) demonstrate the distribution of different elements (Fe, Ga, and In) on its surface, as shown in **Figure** **2**a. The SEM image showed that Fe particles were dispersed in the mixture but with slight agglomeration and that the Fe particles were coated with the oxide layer on the surface. Besides, the Fe elements were evenly distributed in the EGaIn matrix from the EDX images.

In order to demonstrate the outstanding adhesive selection of Fe‐EGaIn on the surface of fructose than PVA, three experiments were carried out. The contact angle of Fe‐EGaIn droplets on PVA film was about 123°, while on the fructose layer was about 79° (Figure 2b). Since the lower contact angle indicated better wettability, the wettability of Fe‐EGaIn droplets on fructose was apparently higher than that on PVA film. Further, a Fe‐EGaIn droplet was placed on an inclined plate (tilt angle of 51°) covered with PVA film, where the droplet could not remain stable and then roll away. On the other hand, the Fe‐EGaIn droplet was tightly adhered on the plate with fructose even on the maximum tilt angle of 90°, as shown in Figure 2c, which clearly demonstrated the better adhesive behavior. Finally, a push‐and‐pull method was introduced to characterize the adhesive force of Fe‐EGaIn on PVA and fructose (Figure S7, Supporting Information). During the test, the probes (covered with PVA film or fructose, 6 mm × 3 mm) were first moved toward the Fe‐EGaIn mounting into its surface. The inserting depth was set as 2 mm for all the cases, and the inserting speed was set as 0.5 mm s^−1^. After reaching the maximum insertion depth, the probes were retracted. Due to the adhesive force of Fe‐EGaIn on PVA and fructose, the probes stayed in contact with the Fe‐EGaIn surface when they were pulled out from the liquid level. Finally, the probes disengaged from the Fe‐EGaIn. The pulling speed of the probes was set as 0.05 mm s^−1^. In this process, the force acting on the probes was recorded by a dynamometer, and the maximal force (the pulling force of probes disengaging from Fe‐EGaIn) was selected as the adhesion force. Figure S8 (Supporting Information) presented the F‐Position curves of Fe‐EGaIn on PVA and fructose during the push‐and‐pull test using the same setting. The maximal force (24.11 mN) on the probe covered with PVA film was much smaller than that with fructose (48.07 mN), as shown in Figure 2d. Above all, these results indicated that the Fe‐EGaIn exhibited significant differences in adhesion on PVA film and fructose, which could be adopted to fabricate liquid metal electronics on PVA films, as shown in Figure S9 (Supporting Information).

In order to reveal the principle of the adhesion differences on PVA film and fructose, the acting functional groups of the PVA film (blue line), fructose (green line), and fructose covered with Fe‐EGaIn oxide layer (orange line) were determined by infrared spectroscopy as shown in Figure 2e,f. Figure 2e showed the molecular structure and acting functional groups of PVA, and there was a small quantity of hydroxyl group (‐OH), which suggested weak hydrogen bond interaction. Contrary to it, there was a large quantity of hydroxyl group in fructose (Figure 2f), which could form strong hydrogen bond with Fe‐EGaIn oxide layer (Ga_2_O_3_), as shown in Figure 2g. Besides, the infrared spectroscopy of fructose covered with Fe‐EGaIn oxide layer showed that there was only an absorption peak shift of the hydroxyl group, which indicated the existence of hydrogen bonds and no new bonds were formed.

### 2.3 Surface Characterization of Fe‐EGaIn

Figure S10 (Supporting Information) showed the surface profile curve of the Fe‐EGaIn layer, which had a maximum height of 56 µm. To assess the electrical performance of the Fe‐EGaIn lines on PVA films, the resistance of different width of Fe‐EGaIn lines after twisting and bending was characterized, respectively. As shown in Figure 2h, the resistance of Fe‐EGaIn lines (line length of 8 cm) increased gradually with the decrease of the lines width (*n* = 4). In the twisting experiment, the Fe‐EGaIn line (width of 1 mm, length of 8 cm) was subjected with twisting angles from 0° to 180° (Figure 2i). The results showed that the twisting experiment did not significantly affect the conductivity. In the bending test, the Fe‐EGaIn line (width of 1 mm, length of 8 cm) was bent with bending radii ranging from 1 to 9 mm (Figure 2j). It was obvious that the resistance did not change obviously, and there was slight increase (Δ*R* = 0.1 Ω) under the bending radii of 1 mm.

### 2.4 Fe‐EGaIn Enabled Magnetic Self‐Healing Electronics

**Figure 3:**
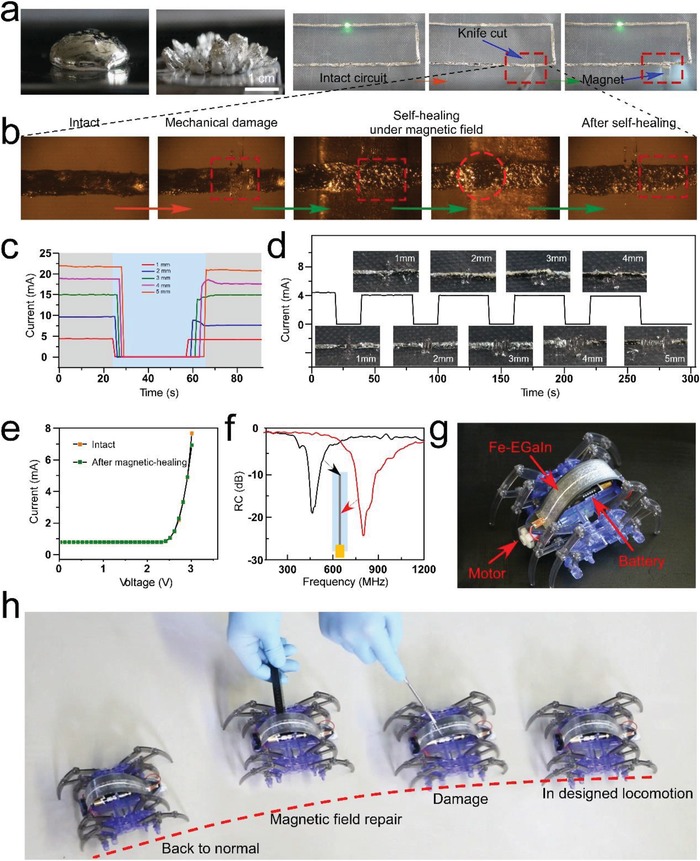
Fe‐EGaIn enabled magnetic self‐healing electronics. a) The photos about magnetic response of the Fe‐EGaIn droplet under a permanent magnet. b) The micrographs of the magnetic healing process of Fe‐EGaIn lines. c) The current variation of different widths of Fe‐EGaIn lines (from 1 to 5 mm) under three conditions (before cut off, cut off, and after healing). d) The current variation and photos of the Fe‐EGaIn line with gaps of different lengths (from 1 to 5 mm) before/after healing. e) The current–voltage (*I*–*V*) curves of the Fe‐EGaIn lines connected with a LED light before/after the magnetic healing. f) The frequency‐dependent measured reflection coefficients of the reconfigurable antenna before/after the magnetic healing. g) A crawling robot with magnetic healing Fe‐EGaIn line. h) The magnetic healing process of the robot from the top‐down view.

As presented in **Figure** **3**a, a typical magnetic response of the Fe‐EGaIn droplet was obvious under a permanent magnet (NdFeB bar, 4 cm in diameter, *B* = 0.4 T). The Fe particles in EGaIn were trapped and distributed in the direction of the magnetic field. Figure 3b shows the magnetic healing process of Fe‐EGaIn lines after a mechanical cut damage, further reconnecting a LED circuit. A Fe‐EGaIn line was cut off using a knife to mimic mechanical damage. Then, a magnet (NdFeB bar, 5 mm × 5 mm, *B* = 0.1 T) was put on the bottom of PVA film to provide magnetic energy. Though moving the magnet from left to right side of the cut gap, the Fe‐EGaIn would follow the movement of the magnet, fill in the gap and fulfill self‐healing process. Finally, removing the magnet and the Fe‐EGaIn line was repaired (Video S1, Supporting Information). The micrographs in Figure 3b showed the enlarged view of the repairing process of Fe‐EGaIn at the gap. It was found that most of Fe‐EGaIn conductive ink was gathered around the edge of the magnet due to the highest magnetic field intensity in this place and that iron particles carried the mixture along the movement of the magnet. The structure of the conductive line after self‐healing showed no difference with the intact conductive line. The magnetic healing time from a single knife cut was about 3 s. The magnetic healing from multipoint damage (3 mechanical damages) was also demonstrated and the healing time was within 10 s. Besides, the magnetic healing ability for different widths of Fe‐EGaIn lines was further tested, as shown in Figure S11 (Supporting Information). Here, different widths of Fe‐EGaIn lines (from 1 to 5 mm) were cut off using the same knife, and repaired via the same magnet (NdFeB bar, 5 mm × 5 mm, *B* = 0.1 T). The curves in Figure 3c showed the current variation of these Fe‐EGaIn lines under three conditions (before cut off, cut off, and after healing). The results showed that different widths of Fe‐EGaIn lines could be repaired in a very short time (less than 10 s). The current was almost back to the original level. As the increases of line width, more Fe‐EGaIn was attracted to the gap and the healing would be much easier with shorter healing time (Figure 3c). Besides, mechanical damage of different lengths (from 1 to 5 mm) was made on a Fe‐EGaIn line (width of 1 mm, length of 10 cm, applied voltage of 5 mV). Magnetic field successfully healed these wide gaps with length less than 5 mm, as shown in Figure 3d. We also found that 5 mm gap exceeded the healing capability of the material. In order to further assess the electrical properties of the healed Fe‐EGaIn lines, the current–voltage (*I*–*V*) curves of the Fe‐EGaIn lines connected with a LED light in Figure 3b were measured. Figure 3e clearly indicated that the resistance variation of the Fe‐EGaIn lines showed no obvious difference after the healing process, which demonstrated the functional recovery of the magnetic self‐healing. For pure EGaIn, it showed similar adhesive difference on PVA and on fructose (Figure S12, Supporting Information). While, the pure EGaIn is not magnetic, the cut conductive line cannot respond under magnetic field or heal by itself due to the high surface tension of liquid metal (Figure S12, Supporting Information). What's more, we encapsulated the Fe‐GaIn circuit in the Ecoflex film to test the self‐healing capability of this system in closed space. After magnetic healing, the Led circuit was enlightened successfully (Figure S13, Supporting Information).

To validate the adaptability of magnetic healing of Fe‐EGaIn electronics, a reconfigurable antenna was manufactured, as shown in Figure 3f. Here, the reconfigurable antenna was linear and its end was connected to the Vector Network Analyzer to measure its frequency‐dependent measured reflection coefficients. According to the expression of antenna resonance frequency (*f* = *c*/2*L*, where *c* is the light velocity in free‐space and *L* is the effective length of the antennas), the resonance frequency of antenna could be tuned by cutting or reconnecting the Fe‐EGaIn line.

The schematic diagrams in Figure 3f shows the original and cut off position of the antenna. After cutting, the resonance frequency of the antenna (red curve) was 797 MHz with the effective length of 18.8 cm. After healing, the resonance frequency of the antenna (black curve) was 465.5 MHz with the original effective length of 32.1 cm. Furthermore, this magnetic healing material also showed application prospect for robotics. To demonstrate its capability of magnetic healing, a Fe‐EGaIn line was placed on the top of a crawling robot to transmit the power signals from the battery to the motor (Figure 3g). Figure 3h showed the magnetic healing process of the robot from the top‐down view (Video S2, Supporting Information). The robot encountered cutting damage from a knife, which cut the power supply and resulted in the cessation of movement. Followed by magnetic healing process with a moving magnet (NdFeB bar, 5 mm × 5 mm, *B* = 0.1 *T*), the robot resumes its motion back to the original route in a very short time.

Recent important studies involving the idea of self‐healing liquid metal materials are usually based on liquid metal–elastomer composite. The mechanism is mainly based on the rupture of liquid metal droplets inside the composite under mechanical damages.^27^ New conductive circuit would be formed around the tearing or puncture parts due to the excellent fluidic property and electrical conductivity of liquid metals. The elastomer with high liquid metal volume fractions (normally ≥50%) would consume a large amount of liquid metal. The resulting elastomer usually with a large thickness would restrict its applications to some extent. What's more, if two conductive lines are pretty close, the self‐healing process would cause the risk of short circuit. In another work, the self‐healing property of the material is realized by sulfur polymer and the healing time is somewhat longer.^44^ In our study, we realize the self‐healing function with the Fe‐GaIn material through magnetic field in a remote way, which is fundamentally different from the elastomers. The resulting conducive line would be much thinner (a thickness of less than 100 µm, Figure S5, Supporting Information) and the consumption of liquid metal is not much. Especially, the healing time is pretty fast within 10 s.

### 2.5 PVA/Fructose Enabled Degradable Electronics

**Figure 4:**
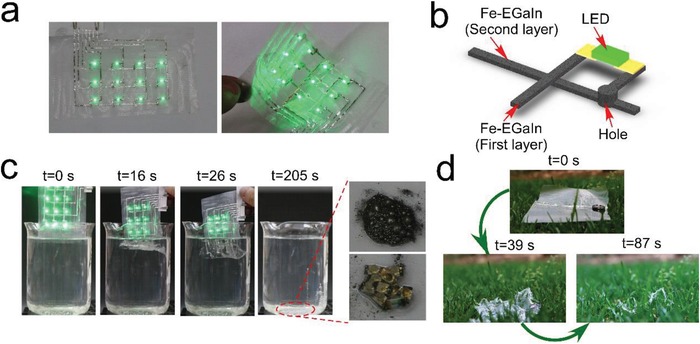
PVA/fructose enabled transient and recyclable electronics. a) The photos of double‐layer LED array. b) Schematic illustration of the structure of the Fe‐EGaIn lines on two layers. c) The dissolution and recycle process of the double‐layer LED array. d) The dissolution process of a LED circuit in the grass.

Till now, transient circuits and recyclable electronics have become a trend, especially in the face of increasing electronic waste in the world, which has become large sources of environmental pollution. Therefore, water‐soluble electronics would serve as one mode for soft and transient electronics with the advantage of easy‐operation. So we chose water‐soluble PVA as substrate. In order to evaluate the practical application effect of degradable electronics, we designed a water dissolution experiment of the flexible double‐layer LED array (**Figure** **4**a). The schematic diagram in Figure 4b showed the structure of the Fe‐EGaIn lines on the two LED layers and some holes, where the Fe‐EGaIn was filled in and connected Fe‐EGaIn lines on the top and bottom layers. It was found that the PVA/fructose film immersed in water (25 °C) dissolved quickly, the Fe‐EGaIn lines were broken and the LEDs were turned off (Figure 4c and Video S3, Supporting Information). The double‐layer LED array was gradually dissolved as the PVA/fructose film gradually immersed under the water. Finally, the PVA/fructose film was completely dissolved in water at 205 s. Later, we collected the LED lights and Fe‐EGaIn droplets in water, and placed them in hydrochloric acid solution (0.1 mol L^−1^). In HCl solution, the oxide layer on the Fe‐EGaIn droplets was removed and the liquid metal mixture would gather into small droplets. In particular, we placed another LED array circuit in the grass to assess the ability of the transient electronics to degrade in the field, as shown in Figure 4d and Video S4 (Supporting Information). As can be seen that this transient electronics could be dissolved in a short time (87 s), when the water was sprayed on it (simulating raining). Besides, the dissolution rate of the PVA/fructose film was related to the temperature of the water. Figure S14 (Supporting Information) shows the dissolution time of PVA/fructose film (10 cm × 5 cm) under different water temperatures. Therefore, we could regulate the dissolution rate of the transient electronics by changing the temperatures of water.

### 2.6 Thermal Transfer Printing

**Figure 5:**
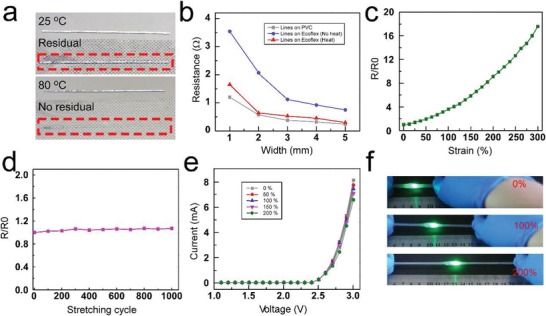
Hydrogen bond break enabled thermal transfer printing. a) The transfer printing of a Fe‐EGaIn line to Ecoflex under different temperature. b) The resistance of Fe‐EGaIn lines printed on the PVA/fructose film and transferred on Ecoflex in 25 and 80 °C. c) The resistance of the stretchable Fe‐EGaIn line under various strains up to 300%. d) The resistance of the stretchable Fe‐EGaIn line under different stretching cycle up to 1000 cycles. e) The *I*–*V* curves of the Fe‐EGaIn lines with LED under various strains (strain 0% to 200%). f) The photographs of the LED lights packaged in Ecoflex under various strains.

Patterning liquid metal on elastomer substrate is an important approach for engineering flexible and stretchable electronics. In our experiments, we found that the adhesive force between Fe‐EGaIn and fructose coating could be decreased in high temperature environment, which might be related to breaking of the hydrogen bond on fructose under high temperature.^45^ **Figure** **5**a showed that there was much residual of Fe‐EGaIn on fructose coating after transfer printed to Ecoflex at room temperature (25 °C), while, almost no residual Fe‐EGaIn at high temperature (80 °C) (Video S5, Supporting Information). Figure 5b showed the resistance of Fe‐EGaIn lines with different widths (length of 8 cm, width from 1 to 5 mm) printed on the PVA/fructose film and transferred on Ecoflex in 25 and 80 °C. The results indicated that the Fe‐EGaIn lines could be transferred to Ecoflex with high efficiency under high temperature, further facilitating to maintain the original electrical performance of the circuits. Using this thermal transfer printing method, we fabricated a stretchable Fe‐EGaIn line (width of 1 mm, length of 5 cm) and measured the resistance under various strains up to 300% (Figure 5c). The mechanical durability of the stretchable Fe‐EGaIn line was also evaluated by a replicated strain test (100% strain) over 1000 times (Figure 5d), and there was no obvious change of the resistance after 1000 cycles. In addition, a LED light was attached to the Ecoflex and connected with the Fe‐EGaIn lines. The *I*–*V* curves (Figure 5e) of the Fe‐EGaIn lines with LED under various strains (0% to 200%) showed that the Fe‐EGaIn lines had remarkable stability and did not affect the normal operation of the circuits (Figure 5f and Video S6, Supporting Information). We also investigated different complex patterns on Ecoflex using the thermal transfer printing method, as shown in Figure S15 (Supporting Information). These results indicated that the stretchable electronics enabled via thermal transfer printing had high conductance stability. To broaden the application scenarios of the transfer printing technology, we covered the 3D curved surface with Ecoflex and then transferred the Fe‐EGaIn line to the 3D curved surface. (Figure S16, Supporting Information) These efforts indicated the capability of the new method.

## 3 Conclusion

In summary, we presented a flexible and multifunctional electronics involving remote magnetic self‐healing, water‐degradable and thermal transfer printing performances, which are enabled by Fe‐GaIn conductive ink, degradable PVA substrate and adhesive fructose, respectively. This soft electronic system was demonstrated with superior electric stability under twisting or bending. The iron microparticles‐doped liquid metal mixture not only work as conductive ink with selective adhesion preference on fructose than on PVA film, but also endow the system with self‐healing capability by remote regulation of a magnet. Under magnetic field, such Fe‐GaIn was found to be accumulated around the places with the highest magnetic intensity and moved along the motion of external magnetic field due to the strong attraction of iron microparticles with the magnet. Both structural repairing and functional recovery were successfully realized in LED circuit with single and multipoint damage. The time of single or multipoint repairment was pretty fast within 10 s. This magnetic healing property of the electronics was also applied for reconfigurable antenna and motion recovery of running robots. Moreover, due to the unique feature of liquid metal and PVA substrate, the fabricated electronic system could dissolve in water and reduce environmental pollution, thus electronic components including Fe‐GaIn materials and other rigid electron parts can be collected and recycled. Through heating, the circuit with Fe‐GaIn ink was demonstrated to thermal transfer printing to other flexible substrates such as Ecoflex with high efficiency, which would facilitate to broaden the application to more stretchable materials. This work provides a novel self‐healing material and technology, which might hold great value for a series of applications in stretchable electronics, recyclable devices and soft robots.

## 4 Experimental Section

*Materials*: Gallium (99.99% purity) and indium (99.99% purity) were purchased from Anhui Minor New Materials Co. Ltd. Fe microparticles (99.5% purity) were purchased from Beijing DK Nano Technology Co., Ltd. The mean diameter of the Fe microparticles is about 10 µm (range: 1–20 µm). The EGaIn (Gallium‐indium eutectic alloys, 75.5 wt% gallium and 24.5 wt% indium) was obtained by stirring gallium and indium together at 80 °C for 1 h. The NdFeB permanent magnets were purchased from Suzhou Abbott magnetic Co. Ltd. The polyvinyl alcohol water‐soluble films (PVA) were purchased from Shanghai Haita plastic technology Co. Ltd. The fructose solution was purchased from Shanghai Rongshi Biotechnology Co. Ltd. The fructose solution printed on PVA films cured at room temperature for 1 min. The Ecoflex 00–30 (Smooth‐On, PA, USA) was prepared by mixing two precursor materials at the weight ratio of 1:1. The mixture was cured at room temperature for 4 h.

*Printing Equipment*: The fructose solution was filled in a ball‐point pen refilled with a diameter of 0.5 mm ball. The fructose solution flowed from the nib as the ball rolled. A mechanical arm (DOBOT Magician, Shenzhen Yuejiang Technology Co. Ltd.) held the ball‐point pen and wrote patterns on PVA films.

*Adhesion Characterization*: The contact angle meter (XG‐CAMC) was used to measure the contact angle of the Fe‐EGaIn droplets on PVA films and on fructose. The adhesion forces of PVA films and fructose with Fe‐EGaIn were measured by the dynamic contact angle measuring instrument (DCAT25). The photos of Fe‐EGaIn droplets rolling off from the tilted slopes were recorded by a camera (Canon EOS 800D).

*Surface Characterization*: The energy‐dispersive X‐ray spectrum (GENESIS) was used to identify the distribution of elements (Fe, Ga, and In) on the Fe‐EGaIn surface. The microscopic images were captured with scanning electron microscopes (S‐4300). An optical profiler (Wyko NT1100, Veeco, USA) was used to measure the height and roughness of Fe‐EGaIn lines printed on PVA substrate. An infrared spectrometer (Horiba Bruker) was applied to determine the acting functional groups of the PVA films and fructose.

*Measurement*: The resistance of Fe‐EGaIn lines was measured using a digital multimeter (Agilent 34420). The frequency‐dependent measured reflection coefficients of the reconfigurable antennas based Fe‐EGaIn lines were measured by the Vector Network Analyzer (Hewlett‐Packard 8753D). The electrical conductivity of the Fe‐EGaIn was measured using a standard four‐point method. The Fe‐EGaIn was filled inside a 750 mm long groove which has a rectangular cross section of 5.1 ± 0.03 × 4.1 ± 0.06 mm.

## Conflict of Interest

The authors declare no conflict of interest.

## Supporting information

Supplementary

Supplementary

Supplementary

Supplementary

Supplementary

Supplementary

Supplementary
