# Supplementary material for: Magnetic Liquid Metal (Fe‐EGaIn) Based Multifunctional Electronics for Remote Self‐Healing Materials, Degradable Electronics, and Thermal Transfer Printing
Source: Adv Sci (Weinh). 2019 Aug 22;6(20):1901478. doi: 10.1002/advs.201901478 (PMC6794621; doi:10.1002/advs.201901478)
Supplement: Supplementary file 1 — Supplementary [file ADVS-6-1901478-s002.pdf]

Copyright WILEY-VCH Verlag GmbH & Co. KGaA, 69469 Weinheim, Germany, 2019.

ADVANCED SCIENCE
Open Access

Supporting Information

for *Adv. Sci.,* DOI: 10.1002/advs.201901478

Magnetic Liquid Metal (Fe-EGaIn) Based Multifunctional Electronics for Remote Self-Healing Materials, Degradable Electronics, and Thermal Transfer Printing

*Rui Guo, Xuyang Sun, Bo Yuan, Hongzhang Wang, and Jing Liu**

Copyright WILEY-VCH Verlag GmbH & Co. KGaA, 69469 Weinheim, Germany, 2019.

Supporting Information

Magnetic liquid metal (Fe-EGaIn) based multifunctional electronics for remote self-healing materials, degradable electronics and thermal transfer printing

*Rui Guo, Xuyang Sun, Bo Yuan, Hongzhang Wang and Jing Liu**

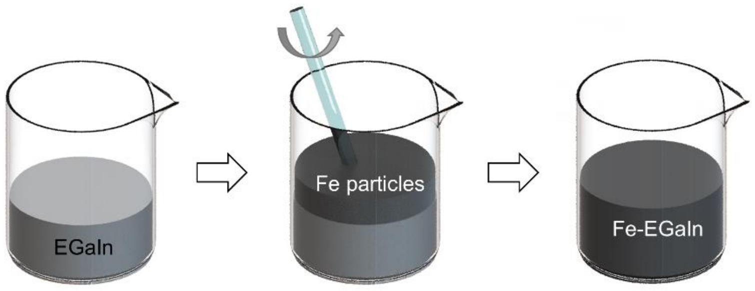

Figure S1 Schematic illustration about the preparation process of Fe-EGaIn.

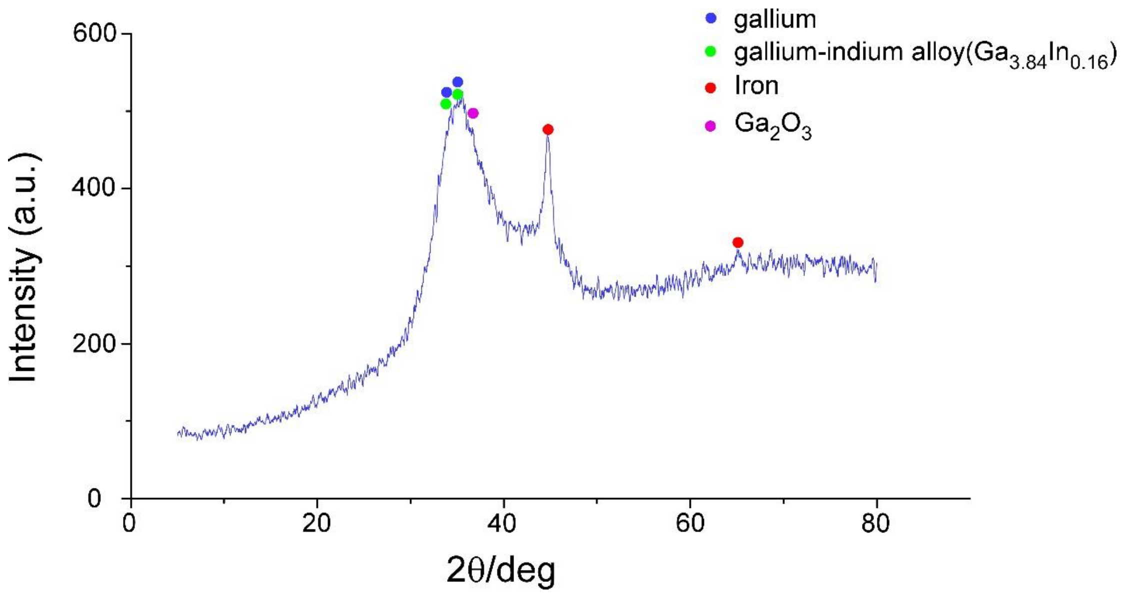

Figure S2 The XRD pattern of the Fe-EGaIn.

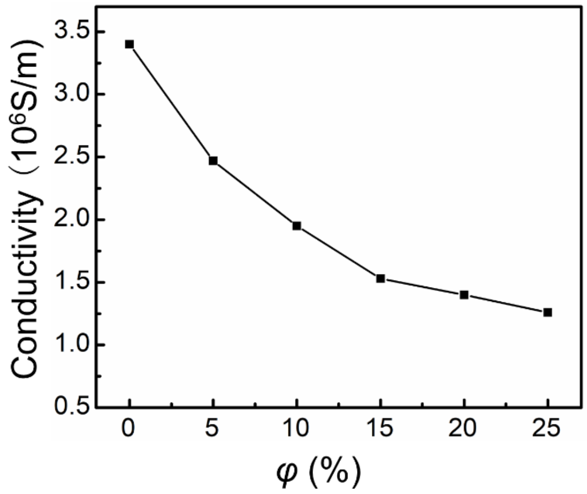

Figure S3 The electrical conductivity of Fe-EGaIn with various packing ratios.

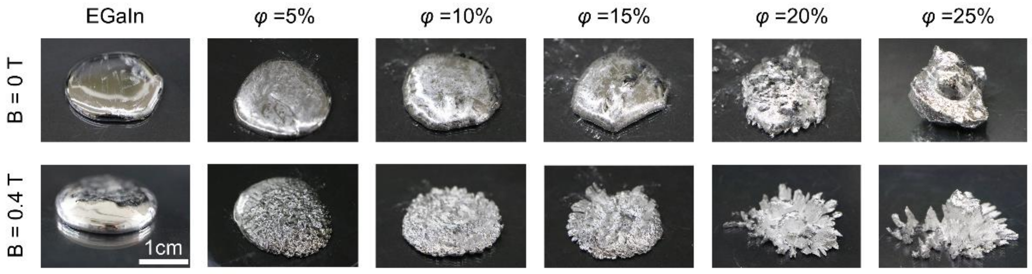

Figure S4 Photos of Fe-EGaIn in various packing ratios with or without magnetic field.

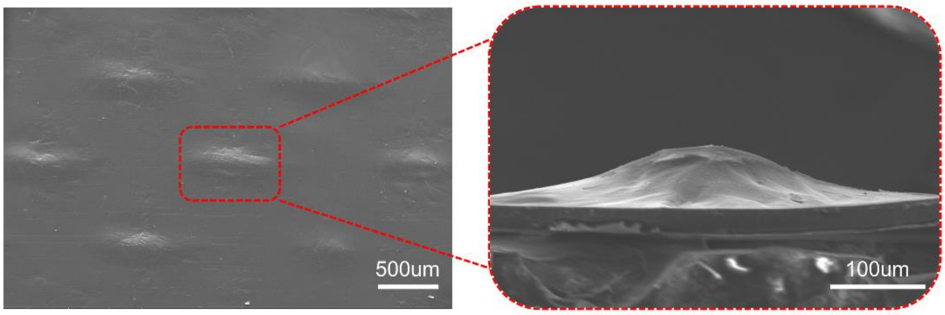

Figure S5 SEM image and cross-sectional SEM image of the bottom layer of a PVA film.

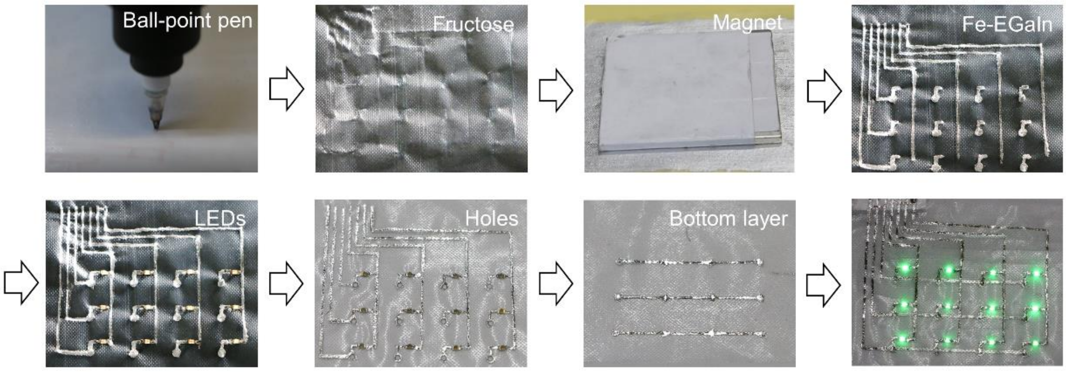

Figure S6 Photos about the preparation process of a double-layer LED array.

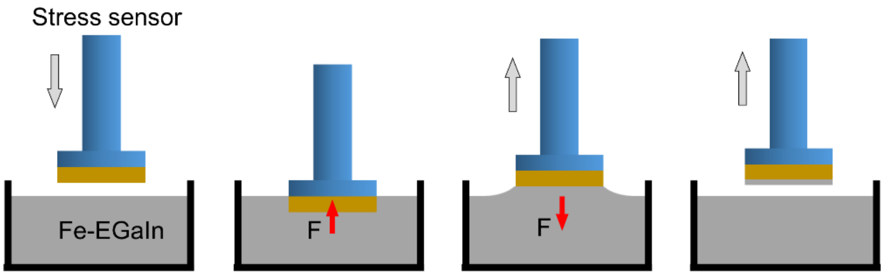

Figure S7 Schematic illustration of the push-and-pull method.

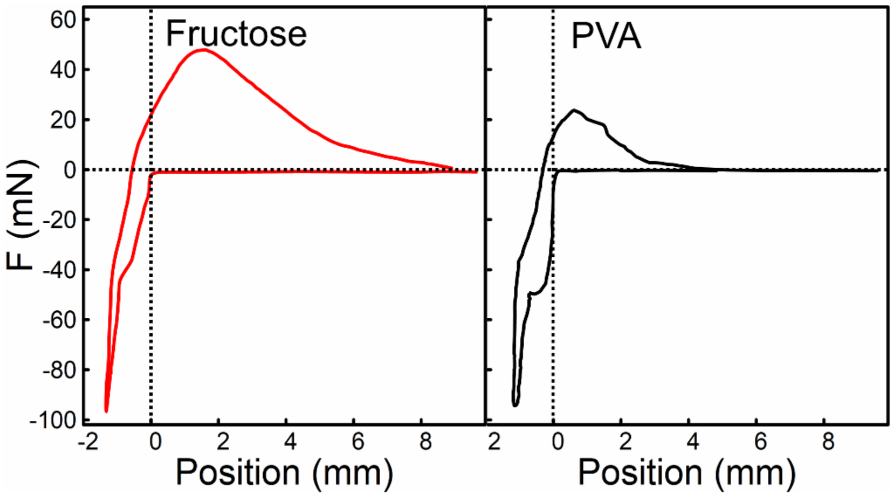

Figure S8 The force curves of PVA and fructose substrates with Fe-EGaIn during the push-and-pull process.

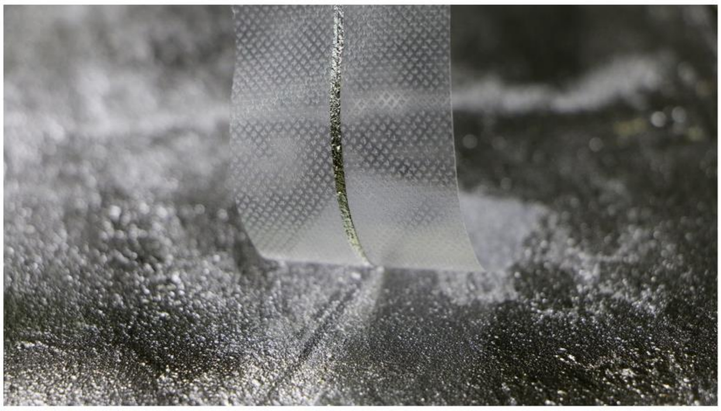

Figure S9 Photograph of the transferred Fe-EGaIn line.

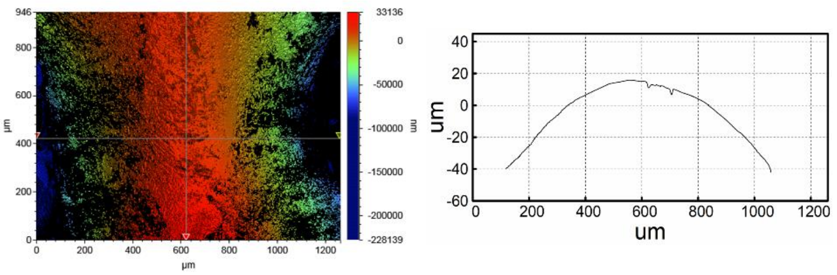

Figure S10 The surface profile of the Fe-EGaIn layer.

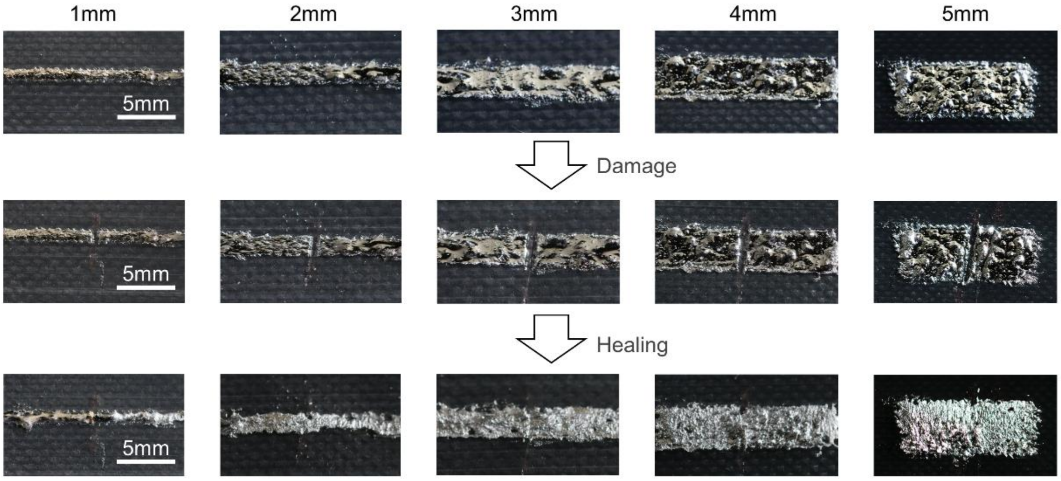

Figure S11 Photos of the Fe-EGaIn lines with different widths before and after magnetic healing.

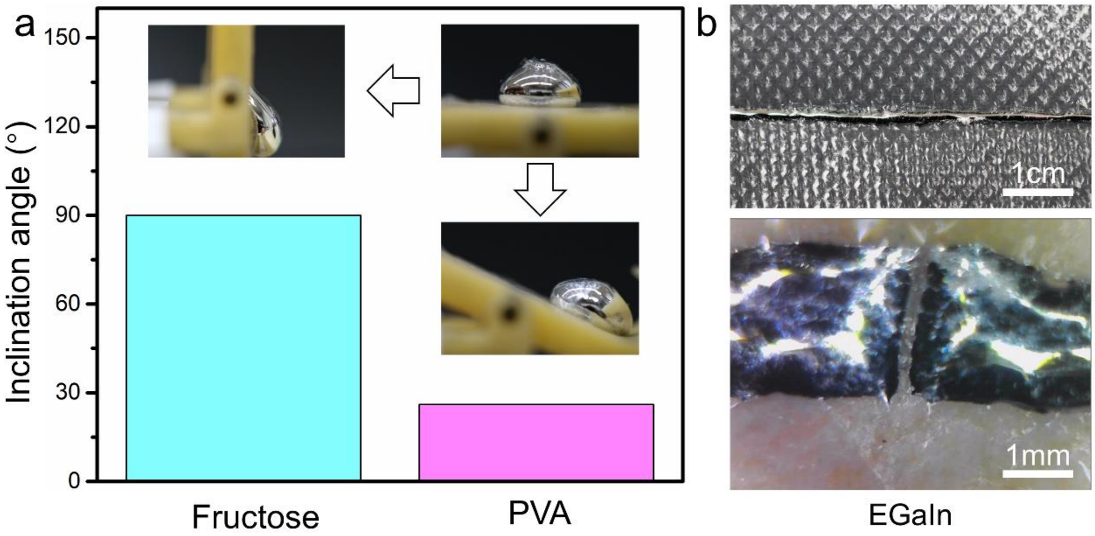

Figure S12 The adhesive property and the self-healing capability of pure liquid metal. (a) The images and roll away angles of EGaIn droplets on inclined plates with PVA and fructose, respectively. (b) EGaIn circuit on PVA film (upper) and the micrograph of the cuting site of pure EGaIn conductive line (lower).

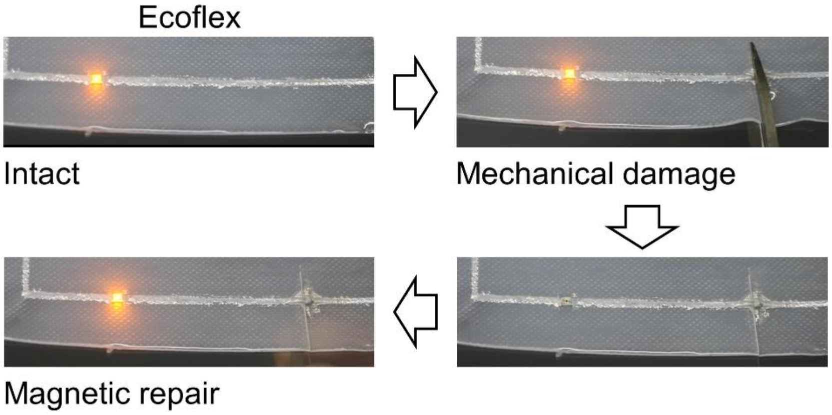

Figure S13 Magnetic repair of circuit in closed sapce.

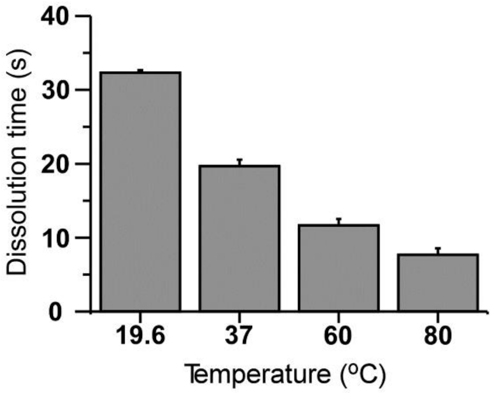

Figure S14 The dissolution time of PVA film (10 cm × 5 cm) under different water temperatures.

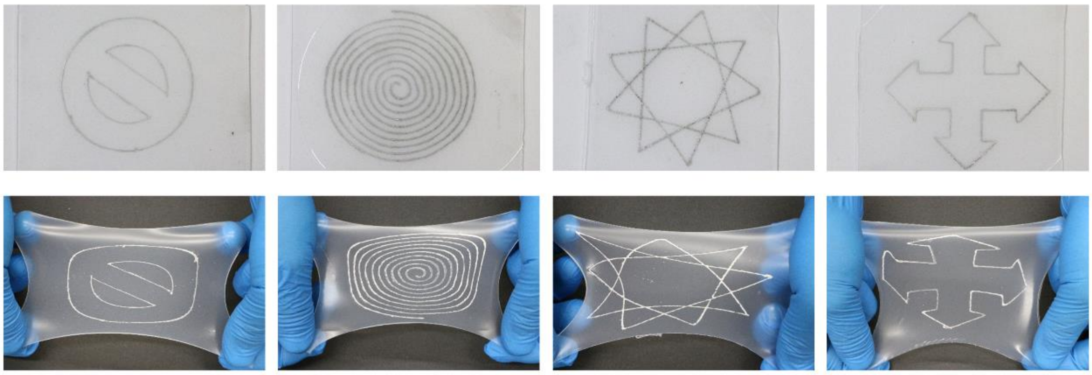

Figure S15 The complex patterns on Ecoflex using the thermal transfer printing method.

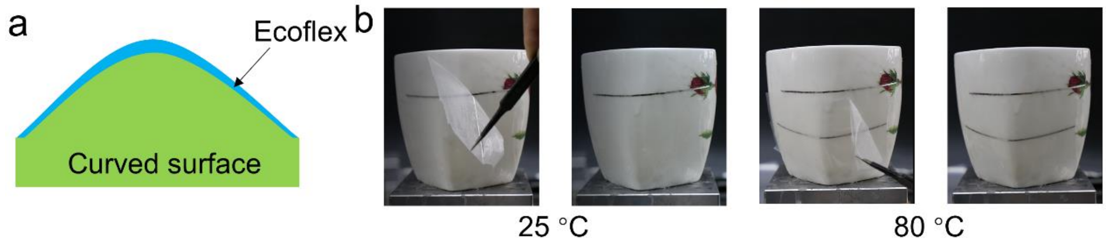

Figure S16 Fe-EGaIn line transferred to 3D curved surfaces via thermal transfer printing technology. (a) Ecoflex on the 3D curved surface. (b) Transferred Fe-EGaIn line to curved surface under different temperature.

Video S1 Fe-EGaIn enabled fast magnetic healing of a LED circuit.

Video S2 The magnetic healing robot.

Video S3 The dissolution of a double-layer LED circuit.

Video S4 The dissolution process of a LED circuit in the grass.

Video S5 Hydrogen bond enabled thermal transfer printing.

Video S6 The LED lights packaged in Ecoflex under various strains.
